# First High-Quality Draft Genome Sequence of *Pasteurella multocida* Sequence Type 128 Isolated from Infected Bone

**DOI:** 10.1128/genomeA.00023-16

**Published:** 2016-03-03

**Authors:** Niloofar Kavousi, Wilhelm Wei Han Eng, Yin Peng Lee, Lian Huat Tan, Ravindran Thuraisingham, Catherine M. Yule, Han Ming Gan

**Affiliations:** a Monash University Malaysia Genomics Facility, Monash University Malaysia, Jalan Lagoon Selatan, Selangor, Malaysia; b School of Science, Monash University Malaysia, Jalan Lagoon Selatan, Selangor, Malaysia; c Sunway Medical Centre, Jalan Lagoon Selatan, Selangor, Malaysia

## Abstract

We report here the first high-quality draft genome sequence of *Pasteurella multocida* sequence type 128, which was isolated from the infected finger bone of an adult female who was bitten by a domestic dog. The draft genome will be a valuable addition to the scarce genomic resources available for *P. multocida*.

## GENOME ANNOUNCEMENT

*Pasteurella multocida* is a Gram-negative coccobacillus and the most common causative microbe isolated from >50% of dog bites and 70% of cat bites (1). *P. multocida* isolates are divided into five groups, A, B, D, E, and F, depending on their capsular antigens (2). Virulence factors, such as capsules, outer membrane proteins, fimbriae, and adhesions, play key roles in the pathogenesis of the disease caused by the bacteria (2). Similar to various pathogens, the capsule and lipopolysaccharide of *P. multocida* allow it to evade host phagocytosis and lysis, leading to severe infection (1, 3).

Following a dog bite on the distal phalanx of her right third finger, a woman developed swelling, redness, and a purulent discharge above the interphalangeal joint. An X ray revealed a lesion consistent with osteomyelitis. Curettage and irrigation of the bone lesion were performed on two occasions, together with a course of antibiotic therapy for 8 weeks, and the wound healed successfully, apart from a loss of joint mobility. A pure laboratory culture was isolated from the infected bone and initially identified as *Pasteurella canis* (strain SMC1) based on biochemical tests. Genomic DNA was extracted from the culture and prepared with Nextera XT (Illumina, San Diego, CA). The library was quantified with Qubit and sequenced on the MiSeq desktop sequencer (2 × 250-bp run configuration) located at the Monash University Malaysia Genomics Facility, Selangor, Malaysia. Raw reads generated from the MiSeq were subsequently adapter trimmed and assembled using Trimmomatic 0.33 and SPAdes 3.5.0, respectively (4, 5). 16S rRNA prediction and *in silico* genome-genome hybridization were performed using RNAmmer and JSpecies 1.2, respectively (6, 7). To determine the sequence type of strain SMC1, its protein-coding genes were predicted with Prodigal version 2.60 and searched against the *P. multocida* Research and Development Corporation (RIRDC) multilocus sequencing type (MLST) database (http://pubmlst.org/pmultocida_rirdc/) hosted at the PubMLST website (http://www.pubmlst.org) (8, 9).

A total of 844,167 paired-end reads were generated for strain SMC1. *De novo* genome assembly produced 23 contigs with total genome length, *N*_50_, G+C content, and coverage of 2,276,171 bp, 202,696 bp, 40.27%, and 70×, respectively. Annotation was done with NCBI Prokaryotic Genome Annotation Pipeline, leading to the prediction of 2,045 protein-coding genes, 10 rRNAs, and 50 tRNAs. The 16S rRNA and whole-genome sequences of strain SMC1 showed 100% similarity and 98.54% average nucleotide identity, respectively, to those of *P. multocida* ATCC 43137^T^, thus confirming its identity as *P. multocida* instead of *P. canis*, which was previously determined based on biochemical tests. Based on *in silico* MLST analysis, the sequence type (ST) of strain SMC1 was found to be ST128. The only other strain of *P. multocida* ST128 that has been reported to date is *P. multocida* strain M25/017, which was isolated from a sheep in New Zealand in 1995 (10).

### Nucleotide sequence accession numbers.

The whole-genome shotgun project for *P. multocida* strain SMC1 has been deposited at DDBJ/EMBL/GenBank under the accession no. LNCO00000000. The version described in this paper is version LNCO01000000.
